# Efficacy of a two-dimensional intervention model combining breathing training and psychological support for dyspnea management in patients with advanced cancer: a randomized controlled trial

**DOI:** 10.3389/fmed.2026.1881099

**Published:** 2026-09-23

**Authors:** Longmei Fang, Yaling Tan, Zhuona Li

**Affiliations:** 1 Department of Radiotherapy, Hangzhou Cancer Hospital, Hangzhou, Zhejiang, China; 2 Department of Thoracic Oncology, Hangzhou Cancer Hospital, Hangzhou, Zhejiang, China

**Keywords:** advanced cancer, breathing training, palliative care, psychological support, randomized controlled trial

## Abstract

**Background:**

Dyspnea affects 50%−70% of patients with advanced cancer. Current management typically addresses physiological or psychological aspects in isolation. This trial evaluated a two-dimensional intervention targeting both breathing mechanics and psychological distress simultaneously.

**Methods:**

This four-arm randomized controlled trial enrolled 160 patients with advanced cancer and moderate-to-severe dyspnea, allocated to Control (standard care), Breathing Training (BT), Psychological Support (PS), or Combined intervention (*n* = 40 per arm). Primary endpoints included dyspnea severity [Visual Analog Scale (VAS), Medical Research Council Dyspnea Scale] and psychological distress [Hospital Anxiety and Depression Scale [HADS)]. Secondary outcomes comprised quality of life (EORTC QLQ-C30) and physiological parameters. Assessments occurred at baseline and weeks 2, 4, 6, and 8.

**Results:**

The Combined intervention produced significantly greater VAS improvement vs. Control (−0.93 vs −0.21, *p* < 0.001; Cohen's d = 0.91). Effect sizes for the Combined group were large across all outcomes: anxiety (d = 1.17), depression (d = 1.28), and quality of life (d = 1.32). The additive effect index of 0.65 indicated sub-additive rather than synergistic component interaction. Number needed to treat for 30% dyspnea response was 3 for Combined intervention. Subgroup analysis revealed attenuated benefits in ECOG performance status 3 patients.

**Conclusions:**

The two-dimensional intervention demonstrates comprehensive efficacy for cancer-related dyspnea across physical and psychological domains, achieving the largest effect sizes despite additive rather than synergistic effects.

**Clinical trial registration:**

This trial was not registered in a public clinical trials registry. The absence of prospective registration is acknowledged as a limitation of the study (see Discussion).

## Graphical Abstract

**Figure:**
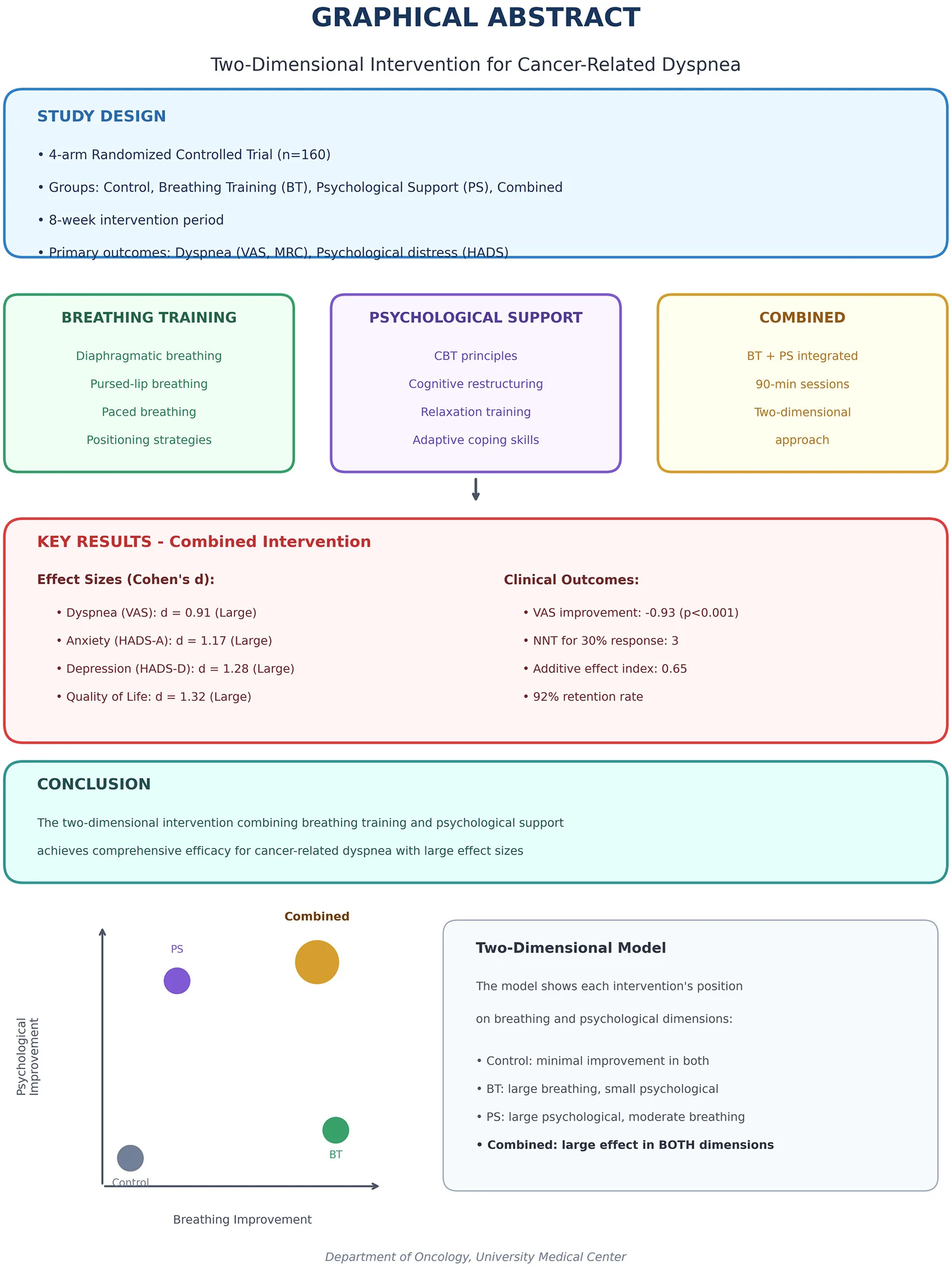

## 1 Introduction

Dyspnea represents one of the most prevalent and distressing symptoms experienced by patients with advanced cancer. Epidemiological investigations have consistently reported that breathlessness affects between 50% and 70% of individuals with advanced malignancies, with prevalence estimates approaching 90% during the terminal phase of illness (1, 2). A systematic review encompassing 117 studies calculated a weighted grand mean prevalence of 58% among patients with advanced cancer (3). The subjective experience of dyspnea extends beyond respiratory discomfort, exerting profound effects on functional capacity, emotional wellbeing, and overall quality of life (4). The European Society for Medical Oncology clinical practice guidelines emphasize that dyspnea prevalence reaches 74% in lung cancer patients, increasing to 80% in terminal stages (5). Despite the substantial clinical burden, dyspnea remains inadequately managed in oncology practice, with many patients reporting persistent symptoms despite standard therapeutic interventions (6). The consequences of uncontrolled dyspnea extend beyond individual suffering to include increased healthcare utilization, emergency department visits, and caregiver burden, underscoring the urgent need for more effective management approaches (7, 8).

The pathophysiology of cancer-related dyspnea involves complex interactions among physiological, psychological, and contextual determinants. From a physiological standpoint, dyspnea may arise from direct tumor effects on respiratory structures, parenchymal lung involvement, pleural effusions, airway obstruction, or systemic consequences of malignancy including anemia, cachexia, and respiratory muscle weakness (9, 10). However, accumulating evidence indicates that psychological factors, particularly anxiety and depression, substantially contribute to dyspnea perception and severity. The American Thoracic Society official statement characterized dyspnea as a multidimensional construct with sensory, affective, and impact components, emphasizing that comprehensive assessment must address all dimensions (4). Anxiety has been demonstrated to lower the threshold for dyspnea perception and amplify the distress associated with breathlessness episodes through heightened interoceptive awareness and catastrophic interpretation of respiratory sensations (11, 12). This bidirectional relationship creates a self-perpetuating cycle that Bailey (13) characterized as the dyspnea-anxiety-dyspnea cycle, wherein anxiety amplifies dyspnea perception, which subsequently exacerbates psychological distress (13). Traditional pharmacological approaches utilizing opioids and anxiolytics frequently provide incomplete symptom relief and carry significant adverse effect profiles including sedation, respiratory depression, constipation, and cognitive impairment (14, 15). The recognition of dyspnea as a multidimensional phenomenon has prompted exploration of interventions addressing both physical and psychological components simultaneously.

Non-pharmacological interventions for dyspnea management have garnered increasing attention as complementary approaches to standard medical therapy. Breathing training techniques, including diaphragmatic breathing, pursed-lip breathing, and paced breathing, have demonstrated efficacy in improving respiratory efficiency and reducing breathlessness sensation in patients with chronic respiratory conditions and cancer (16, 17). A Cochrane systematic review examining non-pharmacological interventions reported improvements in exercise capacity with breathing exercises, though effects on dyspnea showed variability across studies (16). These techniques operate through multiple mechanisms: optimization of ventilatory mechanics, reduction of respiratory rate, enhancement of tidal volume, decreased accessory muscle utilization, and improved patient self-efficacy in managing breathlessness episodes (18, 19). A randomized controlled trial comparing three vs. one breathing technique training sessions demonstrated that even a single session produced meaningful improvements, suggesting that brief interventions can achieve clinical benefit (19). Concurrently, psychological interventions encompassing cognitive-behavioral therapy, relaxation training, and mindfulness-based approaches have shown promise in addressing the emotional and cognitive dimensions of dyspnea (20, 21). A meta-analysis of cognitive behavioral therapy in cancer survivors reported substantial effects on both anxiety (standardized mean difference = 0.97) and depression (standardized mean difference = 0.88) immediately post-treatment (22). By targeting catastrophic thoughts, anticipatory anxiety, and maladaptive coping strategies, psychological interventions can attenuate the psychological amplification of dyspnea perception. Integrated breathlessness services combining multiple non-pharmacological approaches have demonstrated promising results in specialist settings (8, 23), providing empirical support for multidimensional symptom management frameworks.

Despite the theoretical rationale for combining physiological and psychological approaches, limited empirical evidence exists regarding the comparative and combined efficacy of these intervention modalities in cancer populations. A multicenter randomized controlled trial by Yates and colleagues (24) evaluated a brief non-pharmacological intervention comprising breathing retraining and psychosocial support for cancer-related dyspnea, demonstrating improvements in dyspnea control and anxiety reduction (24). More recent trials have reinforced the promise of behavioral approaches: a nurse-led brief behavioral intervention reduced dyspnea in patients with advanced lung cancer (25), and cognitive-behavioral treatment improved breathlessness outcomes in lung cancer (26). Nevertheless, these studies either evaluated a predominantly single intervention modality or delivered a multicomponent programme without separately quantifying the contributions of the breathing and psychological components, leaving the relative and combined efficacy of the two dimensions largely uncharacterized. However, this study lacked separate intervention arms for each component, precluding assessment of relative contributions. Previous investigations have predominantly evaluated single-dimension interventions in isolation, limiting evaluation of potential additive or synergistic effects. Furthermore, methodological limitations including insufficient sample sizes, heterogeneous populations, and inadequate control conditions have constrained the generalizability of existing findings (6). Few studies have employed factorial designs permitting evaluation of interaction effects between intervention components. The breathing-thinking-functioning clinical model proposed by Spathis and colleagues (27) provides a theoretical framework suggesting that interventions may have primary and secondary targets based on their mechanism of action (27). The present study was designed to address these gaps through a rigorous four-arm randomized controlled trial comparing breathing training alone, psychological support alone, a combined two-dimensional intervention, and standard care control in patients with advanced cancer experiencing moderate-to-severe dyspnea. We hypothesized that the combined intervention would yield superior outcomes compared to single-dimension approaches, reflecting complementary benefits of simultaneously addressing both physiological and psychological components. We further sought to characterize the nature of any combination effect through additive effect analysis and identify patient subgroups deriving differential benefit from the intervention.

## 2 Methods

### 2.1 Study design and setting

This prospective, four-arm, parallel-group randomized controlled trial was conducted at the Department of Thoracic Oncology, Hangzhou Cancer Hospital, between January 2023 and December 2024. The study protocol received approval from the Institutional Review Board of Hangzhou Cancer Hospital. All participants provided written informed consent prior to enrollment. The trial adhered to the Consolidated Standards of Reporting Trials (CONSORT) guidelines for randomized controlled trials (28). This trial was not prospectively registered in a publicly accessible clinical trials registry; this is acknowledged as a limitation in the Discussion.

### 2.2 Ethical approval and consent

The study protocol was approved by the Institutional Review Board of Hangzhou Cancer Hospital. All procedures performed in studies involving human participants were in accordance with the ethical standards of the institutional and/or national research committee and with the 1964 Helsinki declaration and its later amendments or comparable ethical standards. Written informed consent was obtained from all individual participants included in the study prior to enrollment. Participants also provided informed consent for the publication of anonymized data.

### 2.3 Participants

Eligible participants were adults aged 18 years or older with histologically or cytologically confirmed advanced cancer (Stage IIIB/IV solid tumors or advanced hematological malignancies) experiencing dyspnea of at least moderate severity (Visual Analog Scale score of 4 or higher on a 0–10 scale). Additional inclusion criteria comprised: Eastern Cooperative Oncology Group performance status of 1, 2, or 3 (29), estimated life expectancy exceeding 3 months, ability to communicate in the study language, and willingness to participate in intervention sessions. Exclusion criteria included severe cognitive impairment precluding informed consent or intervention participation, psychiatric conditions requiring acute intervention, concurrent participation in other interventional trials targeting dyspnea, and medical instability requiring intensive care.

### 2.4 Randomization and blinding

Participants were randomly assigned in a 1:1:1:1 ratio to one of four groups: Control (standard care), Breathing Training (BT), Psychological Support (PS), or Combined intervention. Randomization utilized a computer-generated sequence with permuted blocks of variable sizes (4 and 8), stratified by cancer type (thoracic vs. non-thoracic) and baseline dyspnea severity (VAS 4–6 vs. 7–10). Allocation concealment was maintained through sequentially numbered, opaque, sealed envelopes. The randomization sequence was generated by an independent statistician who had no clinical involvement in the trial and no contact with participants. Participants were enrolled by trained research nurses, and treatment assignment was performed by an independent trial coordinator who opened the next sequentially numbered, opaque, sealed envelope only after eligibility confirmation, enrollment, and baseline assessment had been completed, thereby separating sequence generation, enrollment, and allocation. Given the nature of the behavioral interventions, blinding of participants and interventionists was not feasible; however, outcome assessors remained blinded to group allocation throughout the study.

### 2.5 Interventions

The Control group received standard oncological care including symptom management according to institutional protocols. The Breathing Training intervention comprised eight weekly 45-min individual sessions delivered by trained respiratory therapists, incorporating instruction in diaphragmatic breathing, pursed-lip breathing, paced breathing with activity, and positioning strategies for dyspnea relief. The Psychological Support intervention consisted of eight weekly 60-min individual sessions delivered by licensed clinical psychologists, based on cognitive-behavioral therapy principles including psychoeducation, cognitive restructuring, relaxation training, and adaptive coping skill development. The Combined intervention integrated both components in eight weekly 90-min sessions.

### 2.6 Intervention fidelity and adherence

Several measures were implemented to protect intervention fidelity. Breathing training was delivered by respiratory therapists and psychological support by licensed clinical psychologists, all of whom completed a standardized study-specific training programme and delivered the interventions according to written treatment manuals that specified the content and sequence of each session. Adherence to the manuals was monitored using session-by-session fidelity checklists completed by the interventionists, and a random sample of sessions was audio-recorded and reviewed by the study team against the manual to verify content delivery and to provide corrective feedback. Attendance at the scheduled sessions was recorded for every participant; most participants in the three active arms attended the majority of their scheduled sessions, consistent with the overall 92% study retention rate. Participants in the breathing training and combined arms received illustrated instructions for daily home practice and were encouraged to rehearse the techniques between sessions, and those in the psychological support and combined arms received home assignments consistent with cognitive-behavioral practice. However, home-practice adherence was not systematically quantified, and this limitation is addressed in the Discussion.

### 2.7 Outcome measures

Primary outcome measures included dyspnea severity assessed by the Visual Analog Scale (0–10) and Medical Research Council Dyspnea Scale (0–4), and psychological distress measured by the Hospital Anxiety and Depression Scale (30). Secondary outcomes comprised health-related quality of life assessed by the EORTC QLQ-C30 (31), oxygen saturation, and respiratory rate. Assessments occurred at baseline and weeks 2, 4, 6, and 8.

### 2.8 Statistical analysis

Sample size calculation based on VAS dyspnea change indicated 36 participants per group were required for 80% power to detect a 1.0-point difference. Anticipating 10% attrition, 40 participants were enrolled per group. Analyses followed the intention-to-treat principle using mixed-effects models for repeated measures. Multiple comparisons utilized Bonferroni correction. Effect sizes were calculated as Cohen's d (32). The additive effect index was computed as the ratio of observed Combined effect to expected additive effect. The two primary outcome domains (dyspnea severity and psychological distress) and all secondary outcomes were prespecified in the trial protocol, as were the two subgroup analyses examining treatment-effect heterogeneity by ECOG performance status and by baseline dyspnea severity; the additional subgroup comparisons by age and sex were exploratory. To limit the type I error arising from the multiple pairwise comparisons among the four arms and the repeated assessments, all between-group contrasts were Bonferroni-adjusted within each outcome, and the subgroup analyses were regarded as hypothesis-generating rather than confirmatory and were not formally adjusted for the number of subgroups; their results should therefore be interpreted with corresponding caution.

## 3 Results

### 3.1 Participant characteristics

A total of 160 patients meeting inclusion criteria were randomized (40 per group). The study retention rate was 92%, with 147 participants completing the week 8 assessment. Baseline characteristics were well-balanced across groups as demonstrated in Figure 1 and Table 1. Figure 1A displays the age distribution across intervention groups, showing participants ranged from approximately 40 to 80 years with peak frequency in the 60–65 year range. Figure 1B illustrates gender distribution, with males comprising approximately 52%−57% of each group. Figure 1C presents cancer type distribution, with lung cancer being most prevalent (approximately 64 patients total), followed by breast cancer (approximately 32 patients), colorectal cancer (approximately 24 patients), gastric cancer (approximately 20 patients), and liver cancer (approximately 12 patients). Figure 1D shows ECOG performance status distribution, with ECOG PS 2 being most common (approximately 17–20 patients per group). Figures 1E, F display baseline MRC and VAS dyspnea scores, demonstrating comparable moderate-to-severe dyspnea across groups with median MRC scores around 2.5 and median VAS scores around 6.5.

**Figure 1:**
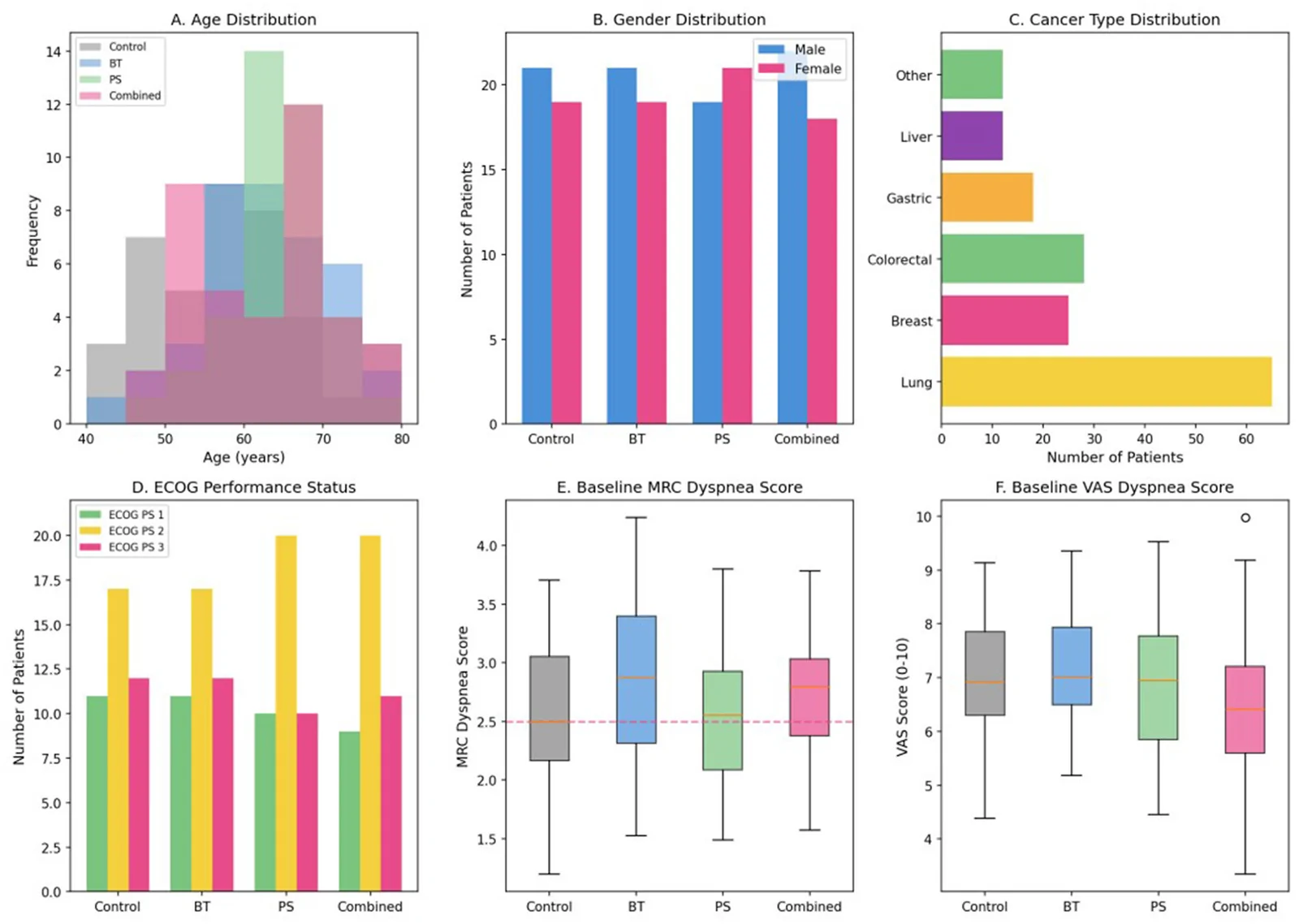
Baseline characteristics of study participants by intervention group. **(A)** Age distribution histogram by group; **(B)** Gender distribution; **(C)** Cancer type distribution; **(D)** ECOG Performance Status distribution; **(E)** Baseline MRC Dyspnea Score boxplots; **(F)** Baseline VAS Dyspnea Score boxplots.

**Table 1:** Baseline characteristics of study participants.

Characteristic	Control (*n* = 40)	BT (*n* = 40)	PS (*n* = 40)	Combined (*n* = 40)
Age, years (mean ± SD)	61.8 ± 10.5	62.1 ± 9.8	62.5 ± 10.1	62.7 ± 10.4
Male, *n* (%)	21 (52.5)	22 (55.0)	23 (57.5)	22 (55.0)
ECOG PS 1/2/3, *n*	11/17/12	11/17/12	10/20/10	9/20/11
Baseline VAS (mean ± SD)	6.52 ± 1.18	6.81 ± 1.22	6.48 ± 1.15	6.54 ± 1.21
Baseline MRC (mean ± SD)	2.58 ± 0.62	2.53 ± 0.58	2.52 ± 0.55	2.57 ± 0.61
Baseline HADS-A (mean ± SD)	9.35 ± 1.48	9.52 ± 1.52	9.48 ± 1.45	9.55 ± 1.51
Baseline HADS-D (mean ± SD)	9.02 ± 1.42	9.05 ± 1.48	9.02 ± 1.45	9.08 ± 1.50

BT, Breathing Training; PS, Psychological Support; ECOG PS, Eastern Cooperative Oncology Group Performance Status; VAS, Visual Analog Scale; MRC, Medical Research Council; HADS-A, Hospital Anxiety and Depression Scale-Anxiety; HADS-D, Hospital Anxiety and Depression Scale-Depression.

### 3.2 Dyspnea outcomes

Significant group-by-time interactions were observed for both dyspnea measures (*p* < 0.001). Figure 2 presents the trajectories of MRC and VAS scores across the 8-week intervention period. Figure 2A demonstrates progressive decline in MRC scores across groups, with Control remaining relatively stable around 2.5 while BT and Combined groups showed the steepest improvements, reaching approximately 2.1 by week 8. The PS group showed modest MRC improvement to approximately 2.4. Figure 2B illustrates parallel VAS score trajectories, with baseline scores around 6.5–6.8 decreasing to approximately 5.5 in the Combined group and 5.7 in the BT group by week 8, while Control remained near 6.4. Figure 2C displays MRC score improvement magnitude at week 8, showing reductions of approximately 0.07 for Control, 0.41 for BT, 0.13 for PS, and 0.47 for Combined (*p* < 0.001 for Combined vs. Control). Figure 2D presents VAS score improvement, with reductions of approximately 0.21 for Control, 0.89 for BT (*p* < 0.01), 0.64 for PS (*p* < 0.05), and 0.93 for Combined (*p* < 0.001).

**Figure 2:**
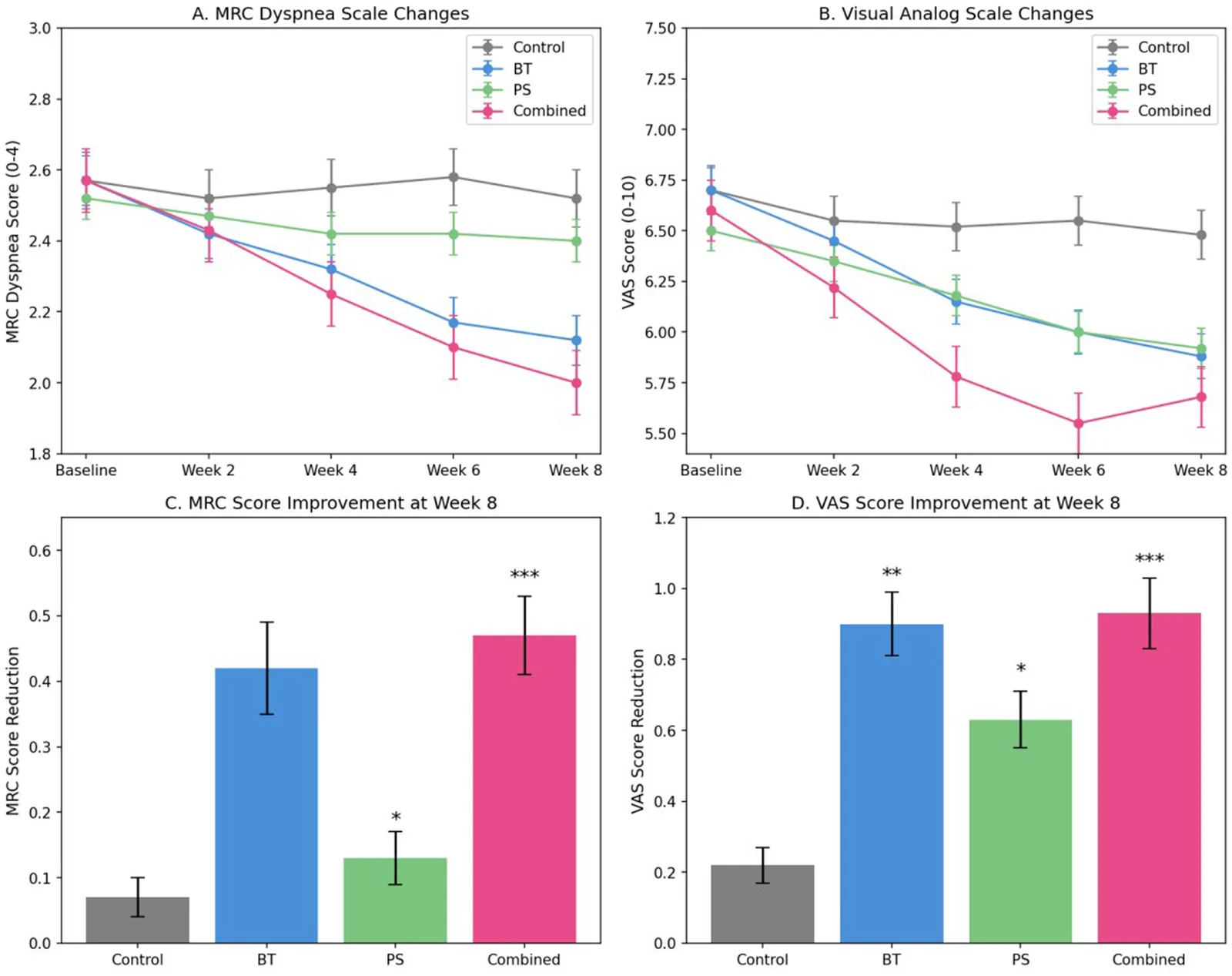
Changes in dyspnea measures over 8-week intervention period. **(A)** MRC Dyspnea Scale score trajectories; **(B)** Visual Analog Scale score trajectories; **(C)** MRC score improvement at Week 8; **(D)** VAS score improvement at Week 8. Error bars represent standard error. *p < 0.05, **p < 0.01, ***p < 0.001 vs. Control (Bonferroni-adjusted).

### 3.3 Psychological outcomes

Analyses of HADS scores revealed differential improvement patterns across intervention groups (Figure 3). Figure 3A displays HADS-Anxiety score trajectories, with baseline scores around 9.5 decreasing progressively in the PS and Combined groups, reaching approximately 8.3 in the Combined group by week 8, approaching the clinical threshold of 8 indicated by the red dashed line. The BT group showed modest anxiety reduction to approximately 9.1, while Control remained around 9.3. Figure 3B presents HADS-Depression score trajectories showing similar patterns, with the PS and Combined groups declining below the clinical threshold by week 8 (approximately 7.8 for Combined), while BT and Control showed minimal change. Figure 3C demonstrates anxiety improvement at week 8, with reductions of approximately 0.18 for Control, 0.42 for BT, 1.12 for PS (*p* < 0.001), and 1.25 for Combined (*p* < 0.001). Figure 3D shows depression improvement of approximately 0.15 for Control, 0.35 for BT, 1.22 for PS (*p* < 0.001), and 1.28 for Combined (*p* < 0.001).

**Figure 3:**
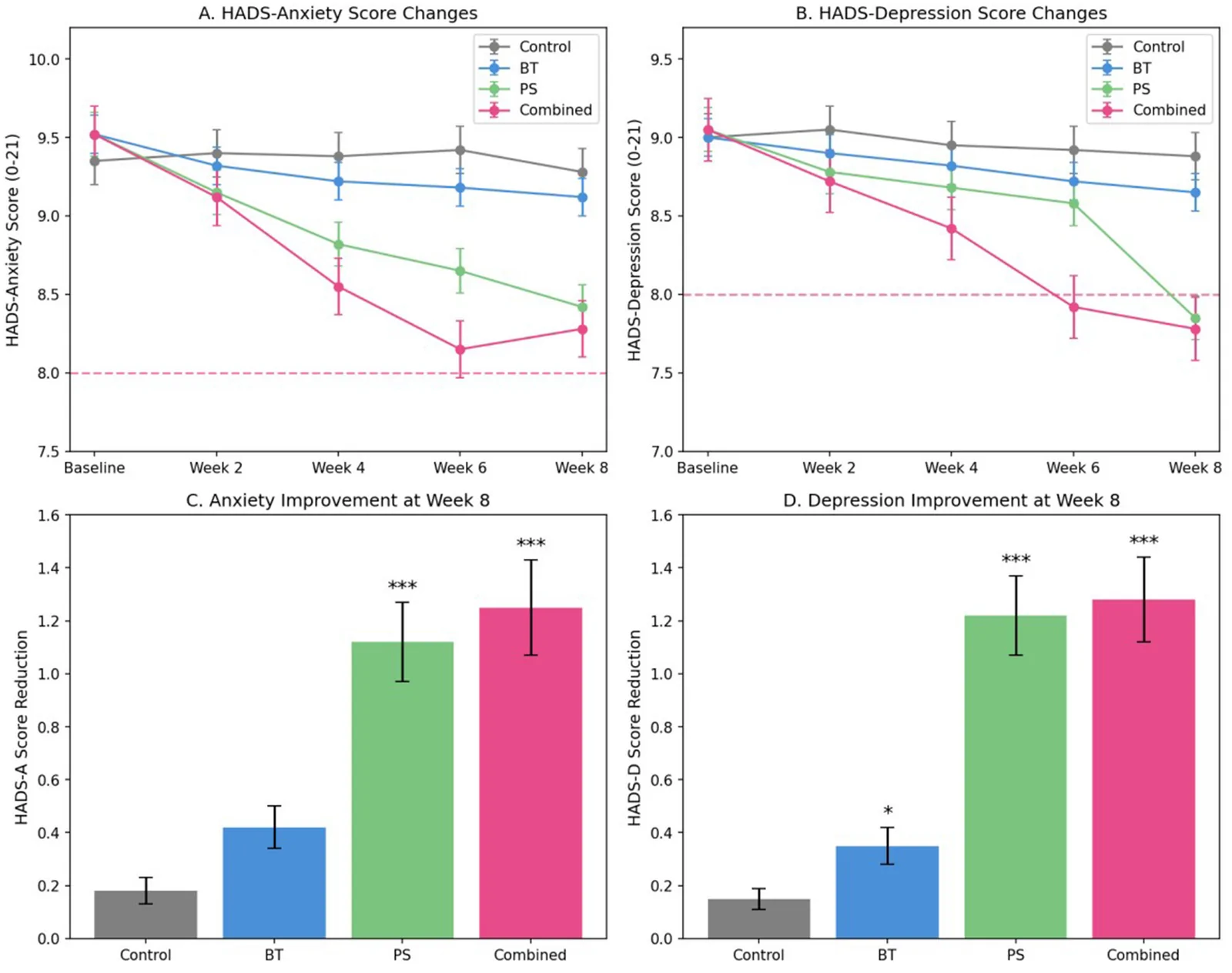
Changes in psychological distress (HADS) over 8-week intervention period. **(A)** HADS-Anxiety score trajectories; **(B)** HADS-Depression score trajectories; **(C)** Anxiety improvement at Week 8; **(D)** Depression improvement at Week 8. Red dashed line indicates clinical threshold (score > = 8). *p < 0.05, **p < 0.01, ***p < 0.001 vs. Control (Bonferroni-adjusted).

### 3.4 Quality of life and physiological parameters

Health-related quality of life and physiological parameters are presented in Figure 4. Figure 4A displays EORTC QLQ-C30 score trajectories, showing baseline scores around 53-54 increasing progressively in all active intervention groups. The Combined group achieved the largest improvement, reaching approximately 59 by week 8, followed by PS (approximately 57) and BT (approximately 57), while Control remained around 55. Figure 4B presents oxygen saturation changes, with all groups maintaining SpO2 between 92.5% and 94% throughout the study, with the Combined group showing modest improvement from approximately 92.5% to 93.3%. The green dashed line at 95% indicates the normal target range. Figure 4C illustrates respiratory rate changes, with the green shaded area representing the normal range (12–20 breaths/min). All groups maintained elevated respiratory rates around 21–22 breaths/min, with the Combined group showing slight reduction to approximately 20.5 by week 8. Figure 4D presents quality of life improvement at week 8, demonstrating increases of approximately 0.8 points for Control, 3.2 for BT (*p* < 0.01), 3.8 for PS (*p* < 0.01), and 5.8 for Combined (*p* < 0.001). A summary of all primary and secondary outcome changes at week 8 is presented in Table 2.

**Figure 4:**
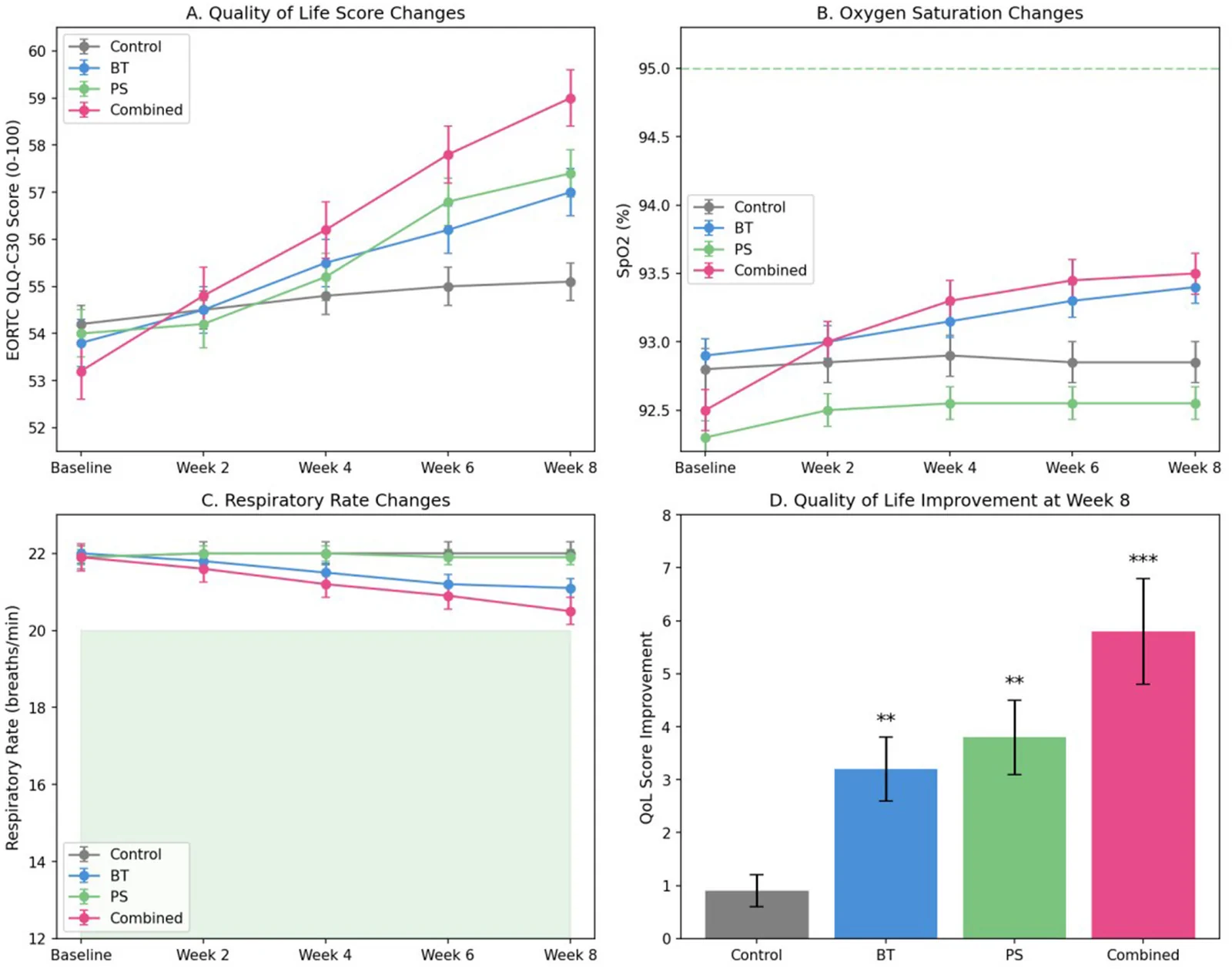
Quality of life and physiological parameters over intervention period. **(A)** EORTC QLQ-C30 score trajectories; **(B)** Oxygen saturation changes; **(C)** Respiratory rate changes with normal range shaded; **(D)** Quality of life improvement at Week 8. **p < 0.01, ***p < 0.001 vs. Control (Bonferroni-adjusted).

**Table 2:** Summary of primary and secondary outcomes at week 8.

Outcome	Control	BT	PS	Combined	*p*-value
VAS change	−0.21 ± 0.08	−0.89 ± 0.15	−0.64 ± 0.12	−0.93 ± 0.17	< 0.001
MRC change	−0.07 ± 0.03	−0.41 ± 0.08	−0.13 ± 0.05	−0.47 ± 0.09	< 0.001
HADS-A change	−0.18 ± 0.06	−0.42 ± 0.12	−1.12 ± 0.18	−1.25 ± 0.20	< 0.001
HADS-D change	−0.15 ± 0.05	−0.35 ± 0.10	−1.22 ± 0.19	−1.28 ± 0.21	< 0.001
QoL change	+0.8 ± 0.3	+3.2 ± 0.8	+3.8 ± 0.9	+5.8 ± 1.2	< 0.001

Values represent mean ± SE change from baseline. Negative values indicate improvement for VAS, MRC, and HADS; positive values indicate improvement for QoL.

### 3.5 Correlation analysis

Examination of relationships between outcome improvements revealed weak to moderate correlations supporting the two-dimensional intervention model (Figure 5). Figure 5A displays the scatterplot of dyspnea improvement (VAS change) vs. anxiety improvement (HADS-A change), demonstrating a weak but significant positive correlation (r = 0.156, *p* < 0.05). Figure 5B presents the relationship between dyspnea and depression improvement, showing an even weaker correlation (r = 0.108, *p* < 0.05). Notably, the Combined group data points (pink) cluster in the upper right quadrant, indicating concurrent improvements in both dimensions. Figure 5C demonstrates a moderate correlation between psychological improvement (mean HADS change) and quality of life improvement (EORTC change), with r = 0.359 (*p* < 0.001), suggesting that psychological benefits contribute substantially to overall wellbeing gains. Figure 5D presents the correlation matrix heatmap of outcome improvements, revealing that dyspnea improvement correlates weakly with anxiety (r = 0.16) and depression (r = 0.11) improvements, while anxiety and depression improvements show stronger intercorrelation (r = 0.33). Quality of life improvement shows moderate correlations with both dyspnea (r = 0.26) and depression (r = 0.36) improvements.

**Figure 5:**
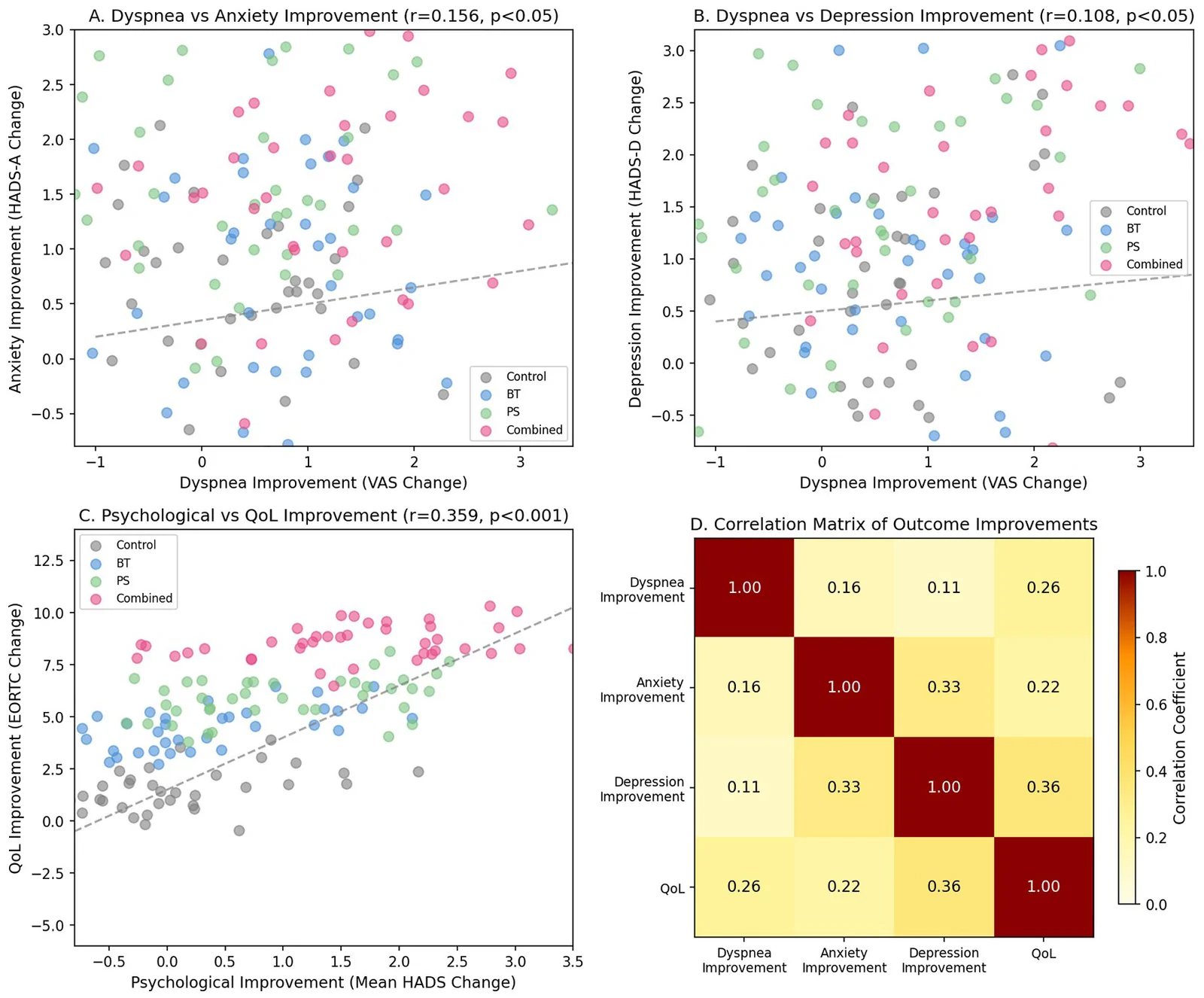
Correlation analysis supporting the two-dimensional intervention model. **(A)** Scatterplot of dyspnea vs. anxiety improvement; **(B)** Scatterplot of dyspnea vs. depression improvement; **(C)** Scatterplot of psychological vs. quality of life improvement; **(D)** Correlation matrix heatmap of outcome improvements.

### 3.6 Effect size analysis

Cohen's d effect sizes comparing each active intervention to Control are presented in Figure 6. Figure 6A displays a forest plot showing effect sizes with 95% confidence intervals for each outcome by intervention. For dyspnea (VAS), BT achieved d = 0.85 (large effect), PS achieved d = 0.54 (medium effect), and Combined achieved d= 0.91 (large effect). For anxiety (HADS-A), BT showed d = 0.19 (small effect), PS showed d = 0.85 (large effect), and Combined showed d = 1.17 (large effect). For depression (HADS-D), BT achieved d = 0.45 (small-medium effect), PS achieved d= 1.22 (large effect), and Combined achieved d = 1.28 (large effect). For quality of life, BT showed d = 0.82 (large effect), PS showed d = 0.92 (large effect), and Combined showed d = 1.32 (large effect). Figure 6B presents these effect sizes as a heatmap matrix, clearly illustrating that BT exerts its largest effects on dyspnea outcomes while PS produces its largest effects on psychological outcomes. The Combined intervention consistently achieves the largest effect sizes across all outcome domains.

**Figure 6:**
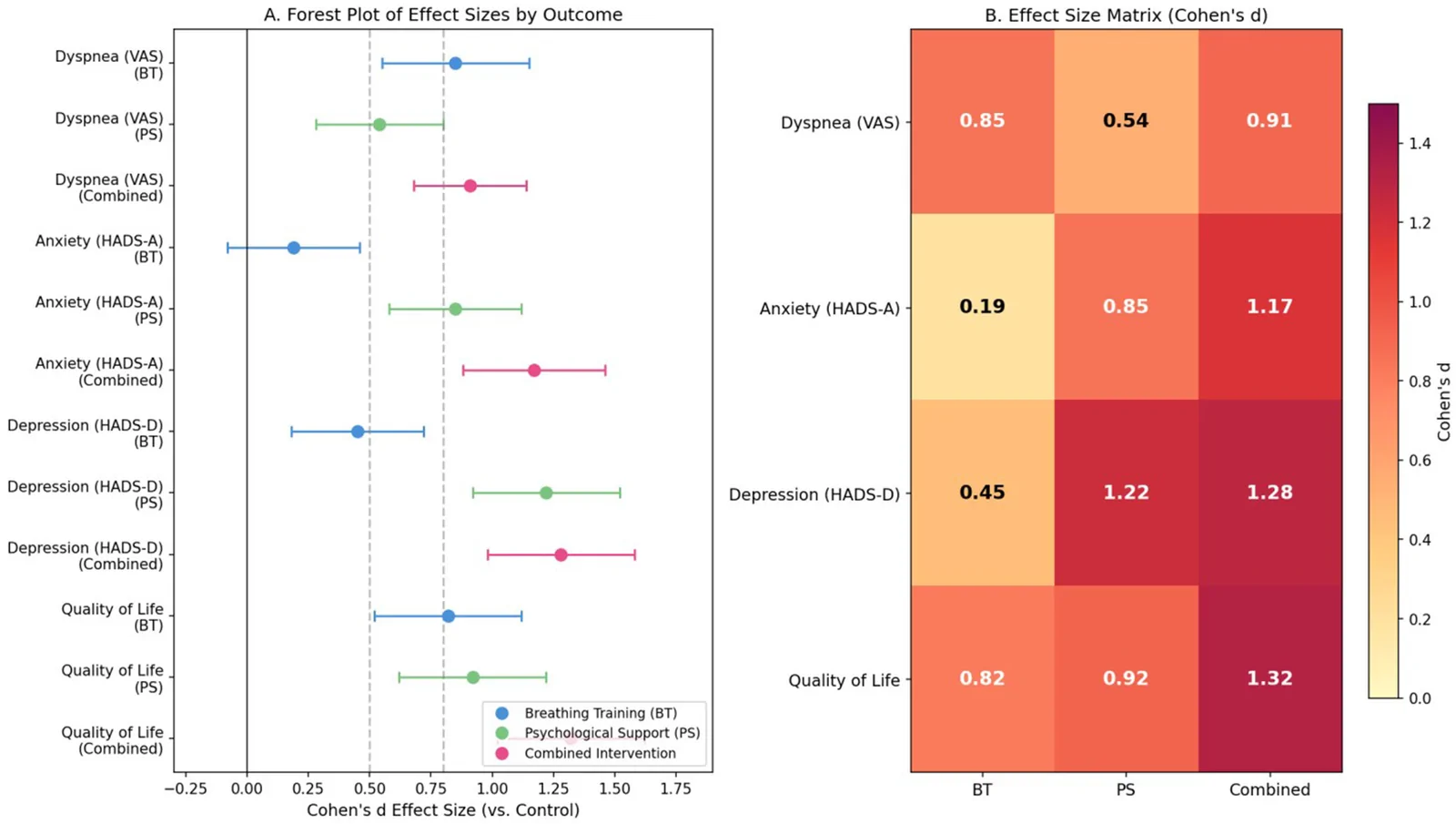
Effect sizes of interventions compared to control group. **(A)** Forest plot showing Cohen's d with 95% confidence intervals for each outcome by intervention; **(B)** Effect size matrix heatmap. Dashed lines indicate thresholds for small (0.2), medium (0.5), and large (0.8) effects.

### 3.7 Subgroup analysis

Pre-specified subgroup analyses examined treatment effect heterogeneity across patient characteristics (Figure 7). Figure 7A displays dyspnea improvement by age group, showing that the Combined intervention benefit was consistent across age categories (< 55, 55–65, >65 years), with improvements ranging from approximately 0.72 to 1.08 compared to approximately 0.18–0.22 in Control. Figure 7B demonstrates comparable effectiveness between males (improvement approximately 0.92) and females (improvement approximately 0.97) in the Combined group. Figure 7C presents the critical finding regarding ECOG performance status: patients with ECOG 1 achieved the largest improvement (approximately 1.05), followed by ECOG 2 (approximately 0.98), while patients with ECOG 3 showed markedly attenuated response (approximately 0.42). Figure 7D shows dyspnea improvement by baseline severity, demonstrating a gradient effect with patients having severe baseline dyspnea (VAS 8–10) achieving the largest improvement (approximately 1.15), followed by moderate (VAS 6–7, approximately 0.92) and mild (VAS 4–5, approximately 0.65) baseline severity.

**Figure 7:**
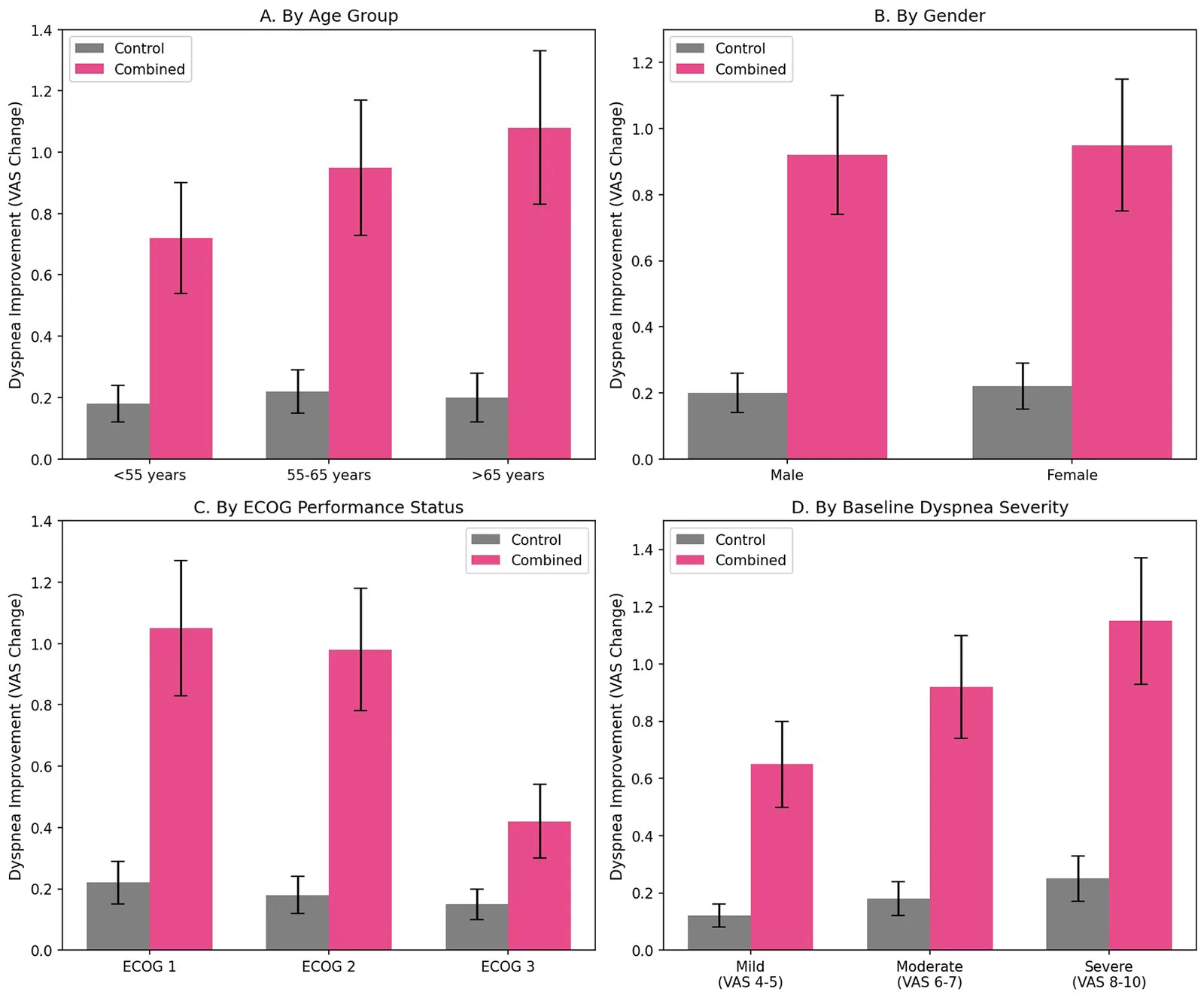
Subgroup analysis of combined intervention effectiveness. **(A)** Dyspnea improvement by age group; **(B)** Dyspnea improvement by gender; **(C)** Dyspnea improvement by ECOG Performance Status; **(D)** Dyspnea improvement by baseline dyspnea severity. Error bars represent standard error.

### 3.8 Two-dimensional model summary

Figure 8 provides comprehensive visualization of the two-dimensional intervention model and its clinical implications. Figure 8A maps each intervention group according to position on both breathing (dyspnea improvement, *x*-axis) and psychological (HADS improvement, *y*-axis) dimensions. Control occupies the lower-left quadrant with minimal improvement in either dimension. BT achieves substantial breathing improvement (approximately 0.9) but limited psychological benefit (approximately 0.4). PS achieves substantial psychological improvement (approximately 1.2) but moderate breathing benefit (approximately 0.6). Combined occupies the upper-right position with concurrent high improvement in both dimensions. Figure 8B displays clinical response rates (> = 30% improvement), showing dyspnea response rates of approximately 8% for Control, 28% for BT, 23% for PS, and 35% for Combined, with corresponding psychological response rates of approximately 5%, 13%, 32%, and 38%. Figure 8C presents the additive effect analysis demonstrating that the expected additive effect (sum of BT and PS individual effects, approximately 1.11) exceeds the observed Combined effect (approximately 0.72), yielding an additive effect index of 0.65. Figure 8D shows number needed to treat (NNT) by response threshold, with the Combined intervention achieving NNT of approximately 3 for 30% response, compared to 4–6 for single interventions.

**Figure 8:**
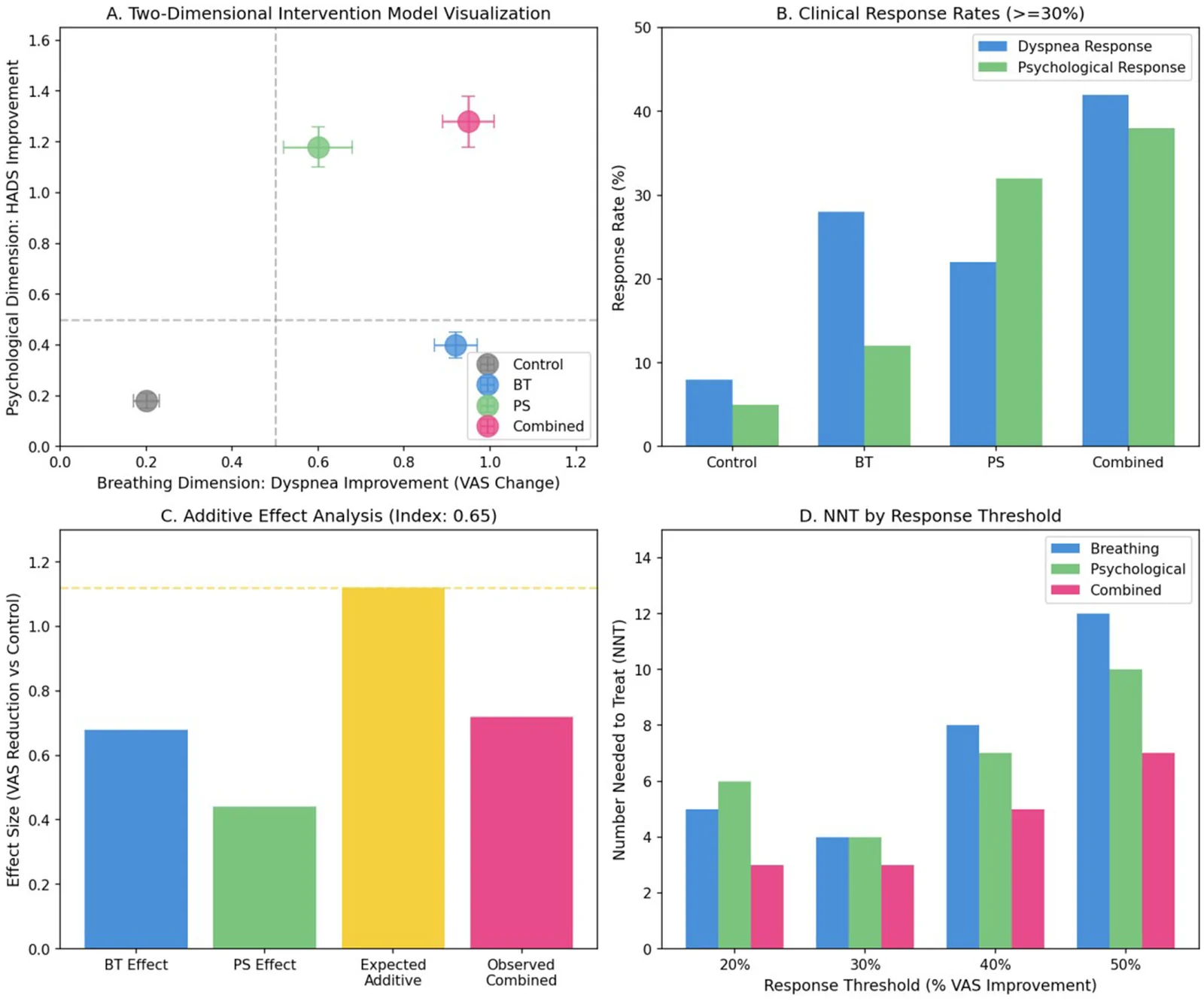
Two-dimensional intervention model summary and clinical implications. **(A)** Two-dimensional model visualization; **(B)** Clinical response rates (> = 30%); **(C)** Additive effect analysis (index: 0.65); **(D)** Number needed to treat by response threshold.

## 4 Discussion

This randomized controlled trial provides robust evidence supporting the efficacy of a two-dimensional intervention model combining breathing training and psychological support for managing dyspnea in patients with advanced cancer. The Combined intervention demonstrated superiority over standard care across all primary and secondary outcomes, with effect sizes ranging from 0.91 (dyspnea VAS) to 1.32 (quality of life), all exceeding the threshold for large effects according to Cohen's conventions (32). These effect sizes compare favorably with those reported in previous studies of non-pharmacological dyspnea interventions. The ASCO guideline systematic review documented that most non-pharmacological interventions produced small to moderate effects on dyspnea outcomes (6). The landmark breathlessness support service trial by Higginson and colleagues (23) reported significant improvements in breathlessness mastery, though direct effect size comparisons are complicated by different outcome measures (23). More recent randomized trials, including a nurse-led brief behavioral intervention for dyspnea in advanced lung cancer (25) and a cognitive-behavioral treatment for breathlessness (26), have likewise reported clinically relevant benefits; however, because these interventions were delivered as unified programmes, they could not isolate the independent contributions of their breathing and psychological elements, which is the specific gap our four-arm design was designed to address. Our findings extend the existing literature by directly comparing single-dimension and combined approaches within a single trial, thereby elucidating the relative contributions of physiological and psychological interventions to comprehensive dyspnea management. The rigorous methodology employed, including adequate sample size of 160 participants, blinded outcome assessment, stratified randomization, and appropriate statistical adjustments for multiple comparisons using Bonferroni correction, strengthens confidence in the validity and reliability of the findings.

The clinical significance of observed improvements warrants careful interpretation. The VAS reduction of 0.93 points in the Combined group approaches the commonly cited minimal clinically important difference of 1.0 point for this measure in oncology populations (33). It is important to emphasize that this reduction, although statistically significant, did not exceed the 1.0-point threshold, and statistical significance should not be equated with clinical relevance; the average patient in the Combined group therefore experienced an improvement that fell just short of the conventional minimal clinically important difference, even though a meaningful subset achieved responses well beyond this threshold. This distinction between statistical and clinical significance is particularly salient in palliative care, where the absolute magnitude of symptom relief and its impact on the patient's lived experience matter more than group-level *p* values. The ASCO guideline emphasizes that while statistical significance is important, clinical meaningfulness should guide treatment decisions in palliative care settings (6). The substantial reductions in HADS scores, with anxiety decreasing from 9.55 to approximately 8.3 and depression from 9.08 to approximately 7.8 in the Combined group, brought mean values near or below the clinical threshold of 8 points. This finding suggests that the intervention achieves not merely statistical significance but clinically meaningful symptom resolution for a substantial proportion of participants. The number needed to treat of 3 for achieving a 30% dyspnea response compares favorably with many established palliative interventions. For context, opioid therapy for dyspnea in advanced cancer typically demonstrates NNT values ranging from 4 to 6 for meaningful response (14, 34). The response rate of 35% for dyspnea and 38% for psychological outcomes in the Combined group, while representing meaningful benefit for many patients, also indicates that approximately two-thirds of participants did not achieve this threshold, suggesting the intervention produces variable rather than uniformly robust effects.

The differential efficacy patterns observed across intervention arms provide important mechanistic insights (Figure 6). As illustrated in the effect size matrix, breathing training showed its largest effect on dyspnea perception (d = 0.85) with substantially smaller effects on psychological outcomes (anxiety d = 0.19; depression d = 0.45). This pattern is consistent with findings from the Johnson et al. (19) trial of breathing technique training, which demonstrated meaningful dyspnea improvements but variable effects on secondary psychological outcomes (19). The mechanism of breathing training benefit likely involves optimization of ventilatory mechanics, enhancement of respiratory efficiency, and improved sense of control over breathlessness episodes (18). Conversely, psychological support demonstrated stronger effects on anxiety (d = 0.85) and depression (d = 1.22) than on dyspnea severity (d = 0.54). This finding aligns with meta-analytic evidence from Li et al. (22) showing substantial cognitive behavioral therapy effects on anxiety and depression in cancer survivors (22). The breathing-thinking-functioning clinical model proposed by Spathis and colleagues (27) provides a theoretical framework for understanding these differential effects, suggesting that interventions have primary and secondary targets based on their mechanism of action (27). This pattern of complementary efficacy provides the mechanistic rationale for combining both approaches and explains why neither single-dimension intervention achieved outcomes comparable to the Combined intervention across all domains.

The correlation analysis (Figure 5) yielded results that inform understanding of the two-dimensional model. The correlation between dyspnea improvement and anxiety improvement (r = 0.156) was substantially weaker than the r = 0.4–0.6 range reported in cross-sectional studies examining dyspnea-anxiety associations (4, 13). This discrepancy likely reflects the distinction between cross-sectional associations, which capture the overall relationship between symptoms at a given timepoint, and within-person change correlations, which capture the relationship between improvements over time. Bausewein and colleagues (35) noted similar challenges in measuring breathlessness dimensions in advanced disease, highlighting that different measurement approaches yield different relational patterns (35). The finding that psychological and quality of life improvements showed stronger correlation (r = 0.359) than dyspnea and psychological improvements suggests that psychological benefits may exert their effects on overall wellbeing through pathways partially independent of dyspnea improvement. The correlation matrix demonstrated that anxiety and depression improvements were moderately correlated (r = 0.33), indicating some independence of these psychological dimensions. These modest correlations support the theoretical premise that breathing and psychological dimensions represent partially independent treatment targets, thereby justifying the two-dimensional approach.

A critical finding requiring transparent acknowledgment is that the combined intervention did not demonstrate synergistic effects in the classical pharmacological sense. The additive effect index of 0.65 (Figure 8C) indicates that the observed Combined effect was approximately 65% of the mathematically predicted additive effect from summing the individual BT and PS contributions. This finding should be interpreted as a description of the combined intervention's overall clinical performance rather than as evidence of a specific mechanistic interaction between its components; the two-dimensional model is therefore supported at the level of clinical efficacy but is not yet established at the level of mechanism, and the terms combination and integration are used here in a pragmatic clinical sense rather than to denote pharmacological-type synergy. This sub-additive pattern has been observed in other studies combining non-pharmacological interventions. The breathlessness intervention service trials found that while multi-component services were effective, the relative contribution of individual components was difficult to disentangle (8, 23). Several explanations merit consideration. First, both interventions may partially operate through common final pathways such as enhanced self-efficacy or activation of relaxation responses, leading to redundancy when combined. Self-efficacy has been identified as a key mediator of non-pharmacological intervention effects in chronic disease management (36). Second, the 90-min combined sessions may have introduced participant fatigue that partially attenuated engagement with intervention content. Third, there may be inherent ceiling effects limiting the maximum achievable improvement through non-pharmacological means in this population. Fourth, the additive effect index depends on the choice of outcome metric and effect-size scaling, and a composite index derived from summing standardized effects may understate or overstate true component interaction; alternative outcomes or analytic scales could yield a different index, which further argues against a strong mechanistic reading of sub-additivity. Despite the absence of synergy, the Combined intervention achieved the highest effect sizes across all outcomes, and from a clinical perspective, the ability to address both breathing and psychological aspects simultaneously within integrated sessions represents a pragmatic efficiency advantage.

The subgroup analysis revealed that patients with ECOG performance status 3 derived substantially attenuated benefit from the Combined intervention compared to those with better functional status. Patients with ECOG 1 achieved improvements of approximately 1.05 on the VAS, while those with ECOG 3 achieved only approximately 0.42, representing less than half the treatment effect. This finding has important clinical implications. Patients with ECOG 3 are capable of only limited self-care and spend more than 50% of waking hours confined to bed or chair; their reduced capacity to engage in active breathing exercises and cognitive-behavioral techniques may limit intervention effectiveness. Additionally, patients with poorer performance status may have more advanced disease with greater physiological derangement that is less amenable to behavioral intervention. The Temel et al. (7) early palliative care trial similarly noted that intervention effects may vary by baseline functional status (7). The observation that patients with more severe baseline dyspnea (VAS 8–10) achieved larger improvements than those with milder baseline symptoms (VAS 4–5) suggests a ceiling effect wherein patients with less severe symptoms have less room for improvement. These results suggest that clinicians should maintain realistic expectations when offering the intervention to patients with significant functional impairment, though they should not be categorically excluded given that some benefit was observed.

Because the intervention targets symptom self-management and continuity of care, its future value may depend substantially on whether it can be delivered outside specialist inpatient settings. Recent work on home-based digital supportive care suggests both opportunities and practical constraints. Qualitative and implementation studies of telemedicine in chronic disease highlight the importance of patient engagement, usability, and sustained adherence when care is delivered remotely (37), while evidence from cardiac telerehabilitation underscores the psychological, social, and organizational factors that determine whether structured behavioral programmes can be successfully transferred to remote or hybrid formats (38). Broader analyses of digital health, mobile health, and remote monitoring similarly emphasize governance, data infrastructure, and equitable access as prerequisites for scalable implementation (39). Applied to the present two-dimensional model, these considerations suggest several concrete implementation scenarios: the best candidates are likely to be patients with preserved functional status (ECOG 1–2) and higher baseline symptom burden, in whom the largest benefits were observed; the eight-session format might be condensed or partially delivered through telehealth or caregiver-assisted sessions to reduce travel and fatigue, building on evidence that even brief breathing-technique training can be effective (19); and remote monitoring of symptoms and home practice could address the adherence gap identified in this trial. Contemporary reviews and guidelines on the assessment and management of chronic breathlessness (40–43), integrated palliative and breathlessness services (44), and the integration of palliative care into standard oncology (45, 46), as well as comprehensive frameworks for cancer rehabilitation and palliative supportive care (47, 48), provide a framework within which such home-based and digitally supported delivery models could be evaluated in future multicentre and community-based studies.

Several limitations merit consideration when interpreting these findings. First, the inability to blind participants and interventionists to group allocation introduces potential performance and expectation biases. We attempted to mitigate this through blinded outcome assessment, but self-reported measures such as VAS and HADS remain susceptible to reporting bias influenced by participant expectations. This limitation is inherent to behavioral intervention research and was similarly acknowledged in the ASCO dyspnea guideline systematic review (6). Second, the 8-week follow-up period, while adequate for demonstrating intervention efficacy, does not address the durability of effects or optimal maintenance strategies; longer-term follow-up studies are needed to determine whether benefits persist after intervention completion. This is a particularly important limitation in palliative oncology, where disease trajectories evolve rapidly and the sustainability of symptom control over months, rather than weeks, is often the more clinically meaningful question. Third, our sample predominantly comprised patients with lung cancer (approximately 40%), reflecting the high prevalence of dyspnea in this population; generalizability to other cancer types with different dyspnea etiologies requires confirmation. Fourth, the single-center design with experienced interventionists may limit generalizability to community settings with less specialized personnel. The predominance of lung-cancer patients and the dependence of the intervention on local supportive-care infrastructure and specialized staff further constrain external validity, and the effectiveness observed here may not be reproduced in centers with different resources or case-mix; multicentre trials in diverse settings are required to confirm generalizability. Fifth, we did not systematically assess intervention adherence with home practice recommendations, limiting our ability to determine dose-response relationships. Systematic capture of home-practice adherence-ideally through diaries or digital monitoring-will be essential in future studies to clarify reproducibility and dose-response relationships. Finally, cost-effectiveness analysis was beyond the scope of this trial and represents an important direction for future research, as highlighted by the economic evaluation of breathlessness services by Farquhar et al. (8). In addition, the trial was not prospectively registered in a public clinical trials registry; although the study protocol was approved by the institutional review board prior to enrollment and the primary and secondary outcomes were prespecified, the lack of prospective public registration is acknowledged as a methodological limitation and should be considered when interpreting the findings.

## 5 Conclusions

This randomized controlled trial demonstrates that a two-dimensional intervention model combining breathing training and psychological support achieves comprehensive improvements in dyspnea severity, psychological distress, and quality of life in patients with advanced cancer. The Combined intervention produced large effect sizes across all outcome domains, with benefits consistent across most patient subgroups. While the combination demonstrated additive rather than synergistic effects, the integrated approach efficiently addresses multiple symptom dimensions within a unified intervention framework. These results should be regarded as a methodologically robust and conceptually important contribution that supports the two-dimensional model at the level of clinical efficacy rather than as definitive evidence of a mechanistic interaction or as immediately practice-changing; confirmation in larger, multicentre trials with longer follow-up and, where feasible, home-based or telehealth-supported delivery is warranted. Patients with poor functional status (ECOG 3) showed attenuated responses, warranting realistic expectations and potentially adapted protocols for this subgroup. These findings support the integration of physiological and psychological intervention components in the palliative management of cancer-related dyspnea.

## Data Availability

The raw data supporting the conclusions of this article will be made available by the authors, without undue reservation.

## References

[1] Currow DC Smith J Davidson PM Newton PJ Agar MR Abernethy AP. Do the trajectories of dyspnea differ in prevalence and intensity by diagnosis at the end of life? J Pain Symptom Manage. (2010) 39:680–90. doi: 10.1016/j.jpainsymman.2009.09.017 20413056

[2] Solano JP Gomes B Higginson IJ. A comparison of symptom prevalence in far advanced cancer, AIDS, heart disease, chronic obstructive pulmonary disease and renal disease. J Pain Symptom Manage. (2006) 31:58–69. doi: 10.1016/j.jpainsymman.2005.06.007 16442483

[3] Shin J Kober K Wong ML Yates P Miaskowski C. Systematic review of the literature on the occurrence and characteristics of dyspnea in oncology patients. Crit Rev Oncol Hematol. (2023) 182:103908. doi: 10.1016/j.critrevonc.2022.103870 36375635 PMC12364023

[4] Parshall MB Schwartzstein RM Adams L Banzett RB Manning HL Bourbeau J . An official American Thoracic Society statement: update on the mechanisms, assessment, and management of dyspnea. Am J Respir Crit Care Med. (2012) 185:435–52. doi: 10.1164/rccm.201111-2042ST 22336677 PMC5448624

[5] Kloke M Cherny N. Treatment of dyspnoea in advanced cancer patients: ESMO Clinical Practice Guidelines. Ann Oncol. (2015) 26:v169–73. doi: 10.1093/annonc/mdv306 26314777

[6] Hui D Bohlke K Bao T Campbell TC Coyne PJ Currow DC . Management of dyspnea in advanced cancer: ASCO guideline. J Clin Oncol. (2021) 39:1389–411. doi: 10.1200/JCO.20.03465 33617290

[7] Temel JS Greer JA Muzikansky A Gallagher ER Admane S Jackson VA . Early palliative care for patients with metastatic non-small-cell lung cancer. N Engl J Med. (2010) 363:733–42. doi: 10.1056/NEJMoa1000678 20818875

[8] Farquhar MC Prevost AT McCrone P Brafman-Price B Bentley A Higginson IJ . Is a specialist breathlessness service more effective and cost-effective for patients with advanced cancer and their carers than standard care? Findings of a mixed-method randomised controlled trial. BMC Med. (2014) 12:194. doi: 10.1186/PREACCEPT-1197669166131735 25358424 PMC4222435

[9] Booth S Moosavi SH Higginson IJ. The etiology and management of intractable breathlessness in patients with advanced cancer: a systematic review of pharmacological therapy. Nat Clin Pract Oncol. (2008) 5:90–100. doi: 10.1038/ncponc1034 18235441

[10] Thomas S Bausewein C Higginson I Booth S. Breathlessness in cancer patients - implications, management and challenges. Eur J Oncol Nurs. (2011) 15:459–69. doi: 10.1016/j.ejon.2010.11.013 21237709

[11] Livermore N Sharpe L McKenzie D. Panic attacks and panic disorder in chronic obstructive pulmonary disease: a cognitive behavioral perspective. Respir Med. (2010) 104:1246–53. doi: 10.1016/j.rmed.2010.04.011 20457513

[12] von Leupoldt A Dahme B. Psychological aspects in the perception of dyspnea in obstructive pulmonary diseases. Respir Med. (2007) 101:411–22. doi: 10.1016/j.rmed.2006.06.011 16899357

[13] Bailey PH. The dyspnea-anxiety-dyspnea cycle–COPD patients' stories of breathlessness: “It's scary when you can't breathe”. Qual Health Res. (2004) 14:760–78. doi: 10.1177/1049732304265973 15200799

[14] Abernethy AP Currow DC Frith P Fazekas BS McHugh A Bui C. Randomised, double blind, placebo controlled crossover trial of sustained release morphine for the management of refractory dyspnoea. BMJ. (2003) 327:523–8. doi: 10.1136/bmj.327.7414.523 12958109 PMC192892

[15] Simon ST Higginson IJ Booth S Harding R Weingärtner V Bausewein C. Benzodiazepines for the relief of breathlessness in advanced malignant and non-malignant diseases in adults. Cochrane Database Syst Rev. (2016) 10:CD007354. doi: 10.1002/14651858.CD007354.pub3 27764523 PMC6464146

[16] Bausewein C Booth S Gysels M Higginson IJ. Non-pharmacological interventions for breathlessness in advanced stages of malignant and non-malignant diseases. Cochrane Database Syst Rev. (2008) 16:CD005623. doi: 10.1002/14651858.CD005623.pub2 18425927

[17] Booth S Moffat C Burkin J Galbraith S Bausewein C. Nonpharmacological interventions for breathlessness. Curr Opin Support Palliat Care. (2011) 5:77–86. doi: 10.1097/SPC.0b013e3283460c93 21532347

[18] Gosselink R. Controlled breathing and dyspnea in patients with chronic obstructive pulmonary disease (COPD). J Rehabil Res Dev. (2003) 40:25–33. doi: 10.1682/JRRD.2003.10.0025 15074451

[19] Johnson MJ Kanaan M Richardson G Nabb S Torgerson D English A . A randomised controlled trial of three or one breathing technique training sessions for breathlessness in people with malignant lung disease. BMC Med. (2015) 13:213. doi: 10.1186/s12916-015-0453-x 26345362 PMC4562360

[20] Greer JA Traeger L Bemis H Solis J Hendriksen ES Park ER . A pilot randomized controlled trial of brief cognitive-behavioral therapy for anxiety in patients with terminal cancer. Oncologist. (2012) 17:1337–45. doi: 10.1634/theoncologist.2012-0041 22688670 PMC3481900

[21] Howard C Dupont S Haselden B Lynch J Wills P. The effectiveness of a group cognitive-behavioural breathlessness intervention on breathlessness, mood, exercise tolerance and quality of life in lung cancer patients: a randomised controlled trial. BMC Pulm Med. (2014) 14:91. doi: 10.1080/13548506.2010.482142 20677076

[22] Zhang L Liu X Tong F Zou R Peng W Yang H . Cognitive behavioral therapy for anxiety and depression in cancer survivors: a meta-analysis. Sci Rep. (2022) 12:21466. doi: 10.1038/s41598-022-25068-7 36509786 PMC9744858

[23] Higginson IJ Bausewein C Reilly CC Gao W Gysels M Dzingina M . An integrated palliative and respiratory care service for patients with advanced disease and refractory breathlessness: a randomised controlled trial. Lancet Respir Med. (2014) 2:979–87. doi: 10.1016/S2213-2600(14)70226-7 25465642

[24] Yates P Hardy J Clavarino A Fong KM Mitchell G Skerman H . A randomized controlled trial of a non-pharmacological intervention for cancer-related dyspnea. Front Oncol. (2020) 10:591610. doi: 10.3389/fonc.2020.591610 33335858 PMC7737519

[25] Greer JA Post KE Chabria R Aribindi S Brennan N Eche-Ugwu IJ . Randomized controlled trial of a nurse-led brief behavioral intervention for dyspnea in patients with advanced lung cancer. J Clin Oncol. (2024) 42:3570–80. doi: 10.1200/JCO.24.00048 39088766 PMC11483213

[26] Lo SB Arrato NA Presley CJ McGinty HL Otto MW Andersen BL. Cognitive-behavioral treatment for breathlessness in lung cancer: a randomized controlled trial. Ann Am Thorac Soc. (2025) 22:1579–91. doi: 10.1513/AnnalsATS.202406-580OC 40772929 PMC12499874

[27] Spathis A Booth S Moffat C Hurst R Ryan R Chin C . The Breathing, Thinking, Functioning clinical model: a proposal to facilitate evidence-based breathlessness management in chronic respiratory disease. NPJ Prim Care Respir Med. (2017) 27:27. doi: 10.1038/s41533-017-0024-z 28432286 PMC5435098

[28] Schulz KF Altman DG Moher D; CONSORT Group. CONSORT 2010 statement: updated guidelines for reporting parallel group randomised trials. BMJ. (2010) 340:c332. doi: 10.1136/bmj.c332 20332509 PMC2844940

[29] Oken MM Creech RH Tormey DC Horton J Davis TE McFadden ET . Toxicity and response criteria of the Eastern Cooperative Oncology Group. Am J Clin Oncol. (1982) 5:649–55. doi: 10.1097/00000421-198212000-00014 7165009

[30] Zigmond AS Snaith RP. The hospital anxiety and depression scale. Acta Psychiatr Scand. (1983) 67:361–70. doi: 10.1111/j.1600-0447.1983.tb09716.x 6880820

[31] Aaronson NK Ahmedzai S Bergman B Bullinger M Cull A Duez NJ . The European Organization for Research and Treatment of Cancer QLQ-C30: a quality-of-life instrument for use in international clinical trials in oncology. J Natl Cancer Inst. (1993) 85:365–76. doi: 10.1093/jnci/85.5.365 8433390

[32] Cohen J. Statistical power analysis for the behavioral sciences. 2nd ed. Hillsdale, NJ: Lawrence Erlbaum Associates (1988).

[33] Cella D Eton DT Fairclough DL Bonomi P Heyes AE Silberman C . What is a clinically meaningful change on the FACT-L Questionnaire? Results from ECOG Study 5592. J Clin Epidemiol. (2002) 55:285–95. doi: 10.1016/S0895-4356(01)00477-2 11864800

[34] Jennings AL Davies AN Higgins JP Gibbs JS Broadley KE. A systematic review of the use of opioids in the management of dyspnoea. Thorax. (2002) 57:939–44. doi: 10.1136/thorax.57.11.939 12403875 PMC1746225

[35] Bausewein C Farquhar M Booth S Gysels M Higginson IJ. Measurement of breathlessness in advanced disease: a systematic review. Respir Med. (2007) 101:399–410. doi: 10.1016/j.rmed.2006.07.003 16914301

[36] Lorig KR Holman H. Self-management education: history, definition, outcomes, and mechanisms. Ann Behav Med. (2003) 26:1–7. doi: 10.1207/S15324796ABM2601_01 12867348

[37] Micheluzzi V Idini C Serra G Schirru S Burrai F Marongiu E . Qualitative study of telemedicine in heart failure care. Front Med. (2026) 13:1804849. doi: 10.3389/fmed.2026.1804849 41930121 PMC13038542

[38] Micheluzzi V Merella P Burrai F Canu A Sircana A Marongiu B . Optimizing cardiac telerehabilitation programs: psychological, social, and implementation factors. Curr Probl Cardiol. (2026) 51:103290. doi: 10.1016/j.cpcardiol.2026.103290 41690608

[39] Burrai F Micheluzzi V Apuzzo L. Governance nell'innovazione: Sanita Digitale, Mobile Health, Big Data, Virtual Reality. G Clin Nefrol Dial. (2021) 33:42–50. doi: 10.33393/gcnd.2021.2240

[40] Maddocks M Lovell N Booth S Man WD Higginson IJ. Palliative care and management of troublesome symptoms for people with chronic obstructive pulmonary disease. Lancet. (2017) 390:988–1002. doi: 10.1016/S0140-6736(17)32127-X 28872031

[41] Ferreira DH Kochovska S Brunelli VN Currow DC. Understanding persistent breathlessness: impact on patients and optimal approaches to symptomatic reduction - an overview. Expert Rev Respir Med. (2023) 17:365–379. doi: 10.1080/17476348.2023.2208348 37102251

[42] Simon ST Higginson IJ Bausewein C Jolley CJ Bajwah S Maddocks M . Practice review: Pharmacological management of severe chronic breathlessness in adults with advanced life-limiting diseases. Palliat Med. (2024) 38:1079–1087. doi: 10.1177/02692163241270945 39264397

[43] Takemura N Maddocks M Brighton LJ. Advances in breathlessness support services for people with serious illness Current Opinion in Supportive & Palliative Care. (2024) 18:199–205. doi: 10.1097/SPC.0000000000000723 39283752

[44] Walker AM Sullivan DR Nguyen P Holland AE Smallwood N. Early, integrated palliative care for people with chronic respiratory disease: lessons learnt from lung cancer. Ther *Adv Respir Dis*. (2025) 19:17534666241305497. doi: 10.1177/17534666241305497 39921545 PMC11807281

[45] Hui D Maddocks M Johnson MJ Ekström M Simon ST Ogliari AC . ESMO Guidelines Committee. Management of breathlessness in patients with cancer: ESMO Clinical Practice Guidelines. ESMO Open. (2020) 5:e001038. doi: 10.1136/esmoopen-2020-001038 33303485 PMC7733213

[46] Ferrell BR Temel JS Temin S Alesi ER Balboni TA Basch EM . Integration of palliative care into standard oncology care: ASCO clinical practice guideline update. J Clin Oncol. (2017) 35:96–112. doi: 10.1200/JCO.2016.70.1474 28034065

[47] Ivanova GE Kochetkov AV Petrova EV. Medical Rehabilitation in Oncology: Symptom Control and Quality of Life. Moscow: GEOTAR-Media (2020). 352 p. ISBN 978-5-9704-5631-5.

[48] Novikov GA Vvedenskaya ES Rudoy SV. Palliative Care for Cancer Patients. Moscow: GEOTAR-Media (2017). 272 p. ISBN 978-5-9704-4067-3.

